# Lack of glutamate neurotransmission in melanin-concentrating hormone neurons alters mouse reproduction and metabolism in a sex-specific manner

**DOI:** 10.1186/s13293-025-00742-3

**Published:** 2025-08-06

**Authors:** Bethany G. Beekly, Dania Zeidan, Wenicios F. Chaves, Jonah-Isabella Sta-Monica, Thomas Saunders, Cristina Saenz de Miera, Christian R. Burgess, Carol F. Elias

**Affiliations:** 1 Department of Molecular & Integrative Physiology, University of Michigan, Ann Arbor, MI USA; 2 Department of Psychology, University of Michigan, Ann Arbor, MI USA; 3 School of Applied Sciences, University of Campinas, Limeira, SP Brazil; 4 Department of Internal Medicine, University of Michigan, Ann Arbor, MI USA; 5 University of Michigan Transgenic Animal Model Core, Ann Arbor, MI USA; 6 Michigan Neuroscience Institute, University of Michigan, Ann Arbor, MI USA; 7 Department of Obstetrics and Gynecology, University of Michigan, Ann Arbor, MI USA

**Keywords:** Lateral hypothalamus, Puberty, Metabolism, Reproduction, Energy homeostasis, Glucose tolerance

## Abstract

Melanin-concentrating hormone (MCH) is a neuropeptide important for the regulation of metabolism, sleep, circadian rhythms, and other body functions. MCH neurons contain the excitatory neurotransmitter glutamate, which is released via vesicular glutamate transporter 2 (VGLUT2). The release of glutamate from MCH neurons is known to play a role in metabolism in males, but its action in female physiology has not been determined. Furthermore, little is known about the role of glutamate release from MCH neurons in reproductive function. We developed a new transgenic mouse model that expresses Cre recombinase driven by the MCH gene (*Pmch*) and deleted *Vglut2* from the MCH neurons. We then assessed reproductive and metabolic function in both sexes. Females exhibited late-onset leanness, delayed sexual maturation, and increased latency to pregnancy. On a high-fat diet, males showed improved insulin resistance, and both sexes displayed an improved daily pattern of food intake. These findings highlight the importance of considering sex as a relevant biological variable in MCH neuronal function.

**Supplementary Information:**

The online version contains supplementary material available at 10.1186/s13293-025-00742-3.

## Highlights

- *Pmch*^*ΔVglut2*^ female, but not male, mice showed a delay in puberty onset and completion.
- *Pmch*^*ΔVglut2*^ female mice were (HFD)-induced disruption of estrous cycles.
- *Pmch*^*ΔVglut2*^ female mice exhibited late-onset reduced total body mass whereas males showed late-onset reduced fat mass.
- *Pmch*^*ΔVglut2*^ male, but not female, mice on HFD showed reduced insulin resistance compared to controls.
- HFD disrupted the daily pattern of food consumption in control *Vglut2*^*flox*^ male and female mice. This disruption was not observed in *Pmch*^*ΔVglut2*^ mice.

**Supplementary Information:**

The online version contains supplementary material available at 10.1186/s13293-025-00742-3.

## Abstract (structured)

**Background:**

Melanin-concentrating hormone (MCH) neurons contribute to the regulation of a diverse array of physiological functions including glucose and energy homeostasis. MCH neurons express genes involved in the synthesis, packaging, and release of glutamate, the brain’s predominant excitatory neurotransmitter. Deletion of vesicular glutamate transporter 2 (VGLUT2, *Slc17a6* gene) in MCH neurons of male mice results in a hypophagic, hyperactive, lean phenotype. However, the metabolic and reproductive effects of VGLUT2 deletion in females have not been fully addressed.

**Methods:**

Previous studies have utilized *Pmch*-Cre mice generated using a bacterial artificial chromosome (BAC). The loci of insertion for the BAC are random and may fail to recapitulate epigenetic regulation at the native gene locus. Here, we generated a novel knock-in *Pmch*-iCre mouse model and investigated the effects of deleting *Slc17a6* from MCH neurons in both male and female mice. Assessment of sexual maturation, fertility, glucose homeostasis and energy balance (food intake, body composition, energy expenditure, locomotion, glucose and fat oxidation) on regular chow and high fat diet (HFD) were performed in both sexes.

**Results:**

Lack of glutamate neurotransmission in MCH neurons (*Pmch*^*ΔVglut2*^ mice) delays puberty onset and completion in female, but not male mice. *Pmch*^*ΔVglut2*^ females were also protected against (HFD)-induced disruption of estrous cycles. On a regular chow diet, neither male nor female *Pmch*^*ΔVglut2*^ mice showed differences in body weight, food intake, or lean and fat masses compared to littermate controls until 20 weeks of age. At 24 weeks of age, *Pmch*^*ΔVglut2*^ females exhibited reduced total body mass, and males had lower fat mass. *Pmch*^*ΔVglut2*^ female mice also gained less weight on HFD than littermate controls. An attenuation of HFD-induced disruption of daily feeding pattern was observed in *Pmch*^*ΔVglut2*^ mice of both sexes, while only males were protected against insulin resistance on HFD.

**Conclusions:**

Glutamate neurotransmission from MCH neurons has a sex-specific role in sexual maturation, fertility, typical weight gain trajectory, and HFD-induced weight gain and insulin resistance. In addition, a sex-independent role in daily feeding pattern was observed.

**Supplementary Information:**

The online version contains supplementary material available at 10.1186/s13293-025-00742-3.

## Background

Hypothalamic melanin-concentrating hormone (MCH) neurons contribute to the regulation of a variety of physiological systems including glucose homeostasis, energy balance, sleep and motivated behaviors [1–6]. Mice deficient in the prepro-MCH (*Pmch*) gene (MCH-knockout or MCH-KO mice) are hyperactive, hypophagic, and lean, and are also protected from high fat diet (HFD)-induced obesity, while overexpression of *Pmch* induces obesity and insulin resistance [7–10]. Intracerebroventricular administration of MCH and chemogenetic activation of MCH neurons increase food intake in male rats in an MCH receptor 1 (MCHR1) dependent manner [11–13].

MCH neurons express genes involved in the synthesis, packaging, and release of glutamate, the brain’s predominant excitatory neurotransmitter [2, 14–16]. Mice with deletion of vesicular glutamate transporter 2 (VGLUT2, *Slc17a6* gene) in MCH neurons also show a hypophagic lean phenotype and improved glucose tolerance [15, 16]. When the metabolic phenotypes of male MCH-KO, MCH neuron-ablated, and transgenic *Pmch*-Cre;*Slc17a6*^fl/fl^ mice (which lack VGLUT2 in MCH neurons) are compared, functional redundancies between MCH and glutamatergic neurotransmissions are revealed. All three experimental groups exhibit decreased body weight and adiposity, increased locomotor activity, late-onset hypophagia, attenuated weight gain on high-fat diet, and reductions in circulating leptin [15, 16]. On the other hand, specific indicators of glucose sensing and handling differ between groups. For example, MCH neuronal ablation and deletion of *Vglut2* from MCH neurons—but *not* MCH-KO—resulted in reduced sucrose preference and improved glucose tolerance [16]. These findings suggest that at least some of the contributions of MCH neurons to metabolic regulation arise not from MCH peptide itself, but from co-released neurotransmitters, particularly glutamate.

Importantly, the effect of conditional deletion of *Vglut2* from MCH neurons has been mainly assessed in male mice [16–19]. This constitutes a significant gap in the field’s understanding of the MCH system and its role in energy balance, as biological sex and gonadal hormones are closely linked to metabolism. Of note, sex differences in glucose handling are widely described in rodents and primates and the prevalence of type II diabetes and associated comorbidities is dissimilar in men and women [20–22].

The reproductive development and function of mice with conditional deletion of VGLUT2 from MCH neurons have not been investigated despite the close relationship between the neuroendocrine reproductive axis and energy balance. Pubertal timing is intrinsically linked to nutritional status: low body weight and obesity are associated with disrupted pubertal timing [23–26]. Adult fertility is also impaired by both under- and overnutrition and by insulin resistance [27–29]. Furthermore, *Vglut2* deletion in MCH neurons alters daily REM/non-REM sleep patterns, which might also impact reproductive development and function independently of metabolic effects [19, 30–33].

Previous studies have utilized MCH-Cre mice generated using a bacterial artificial chromosome (BAC) containing the coding region for Cre recombinase flanked by upstream and downstream regulatory elements of the *Pmch* gene [16–19, 34]. The use of BAC transgenes was once a common method of introducing DNA sequences to the genome of a model organism. However, BAC integration sites are random. BACs will bring in their own unique set of regulatory elements and cause local rearrangements of chromosomes at integration sites, both of the BAC and the host chromosome. All of this can potentially impose additional layers of regulation on Cre expression and the expression of endogenous genes [35–37]. It also means BAC transgenes may fail to recapitulate epigenetic regulation that occurs at the native gene locus and relies on enhancers that can be located hundreds or thousands of bases up- or downstream [38, 39]. Sexual differentiation, pubertal development, social behaviors, pregnancy, lactation, and reproductive senescence are all associated with epigenetic changes that may rely on enhancer regions outside the limits of the BAC transgenes [40–43].

Such caveats may be of concern when there is an intent to study individuals across the lifespan and during or after life events such as pregnancy. Indeed, previous work in our laboratory has shown that while *Pmch* is transiently expressed in several nuclei of the rostral forebrain at the end of lactation, it does not reliably correlate with Cre expression in the *Pmch*-Cre (BAC) transgenic mouse [44]. Thus, this model may not be an ideal choice to study the role of MCH neurons in female mouse physiology.

To increase opportunities to study the MCH system in both sexes, we generated a knock-in mouse line, ensuring that Cre recombinase expression occurs under the endogenous regulatory control of the *Pmch* gene locus. We also used the sequence for codon-improved Cre recombinase (iCre), which applies mammalian codon usage to the prokaryotic Cre sequence and thus contains numerous base substitutions which do not alter the peptide sequence but do improve Cre expression and reduce the chances of epigenetic silencing in mammals [45].

By crossing the *Pmch*-iCre with *Vglut2*^*flox*^ mice, we generated a new *Pmch*^*ΔVglut2*^ mouse line and assessed the reproductive and metabolic function in both sexes. We reproduced most, but not all, of the published findings in males. Most notably, several sex differences were observed, reaffirming the importance of incorporating biological sex as a variable into neuroscience research, including classical neurotransmission, the MCH system, and the neuroendocrine control of metabolism.

## Methods

### Animal care

Mice were housed in a vivarium at the University of Michigan with a 12:12 light/dark cycle and free access to food and water. Mice were fed with phytoestrogen-reduced Teklad global diet 2016 (16% protein/4% fat, 3.0 kcal/g) or a high-fat diet TD.88,137 (42% calories from fat, 4.5 kcal/g) [46, 47]. During breeding, mice were fed phytoestrogen-reduced Teklad global diet 2019 (19% protein and 8% fat, 3.3 kcal/g) to accommodate additional nutritional needs of pregnant and nursing dams [47, 48]. Phytoestrogen-reduced diet is routinely used in our laboratory to avoid the effects of exogenous estrogens on mouse physiology. All procedures and experiments were carried out in accordance with the guidelines established by the National Institutes of Health Guide for the Care and Use of Laboratory Animals and approved by the University of Michigan IACUC (Animal Protocol # PRO00009706, UM Transgenic Animal Core and PRO00010420, Elias Lab).

### Generation and validation of knock-in *Pmch*-iCre mouse model

**Fig. 1:**
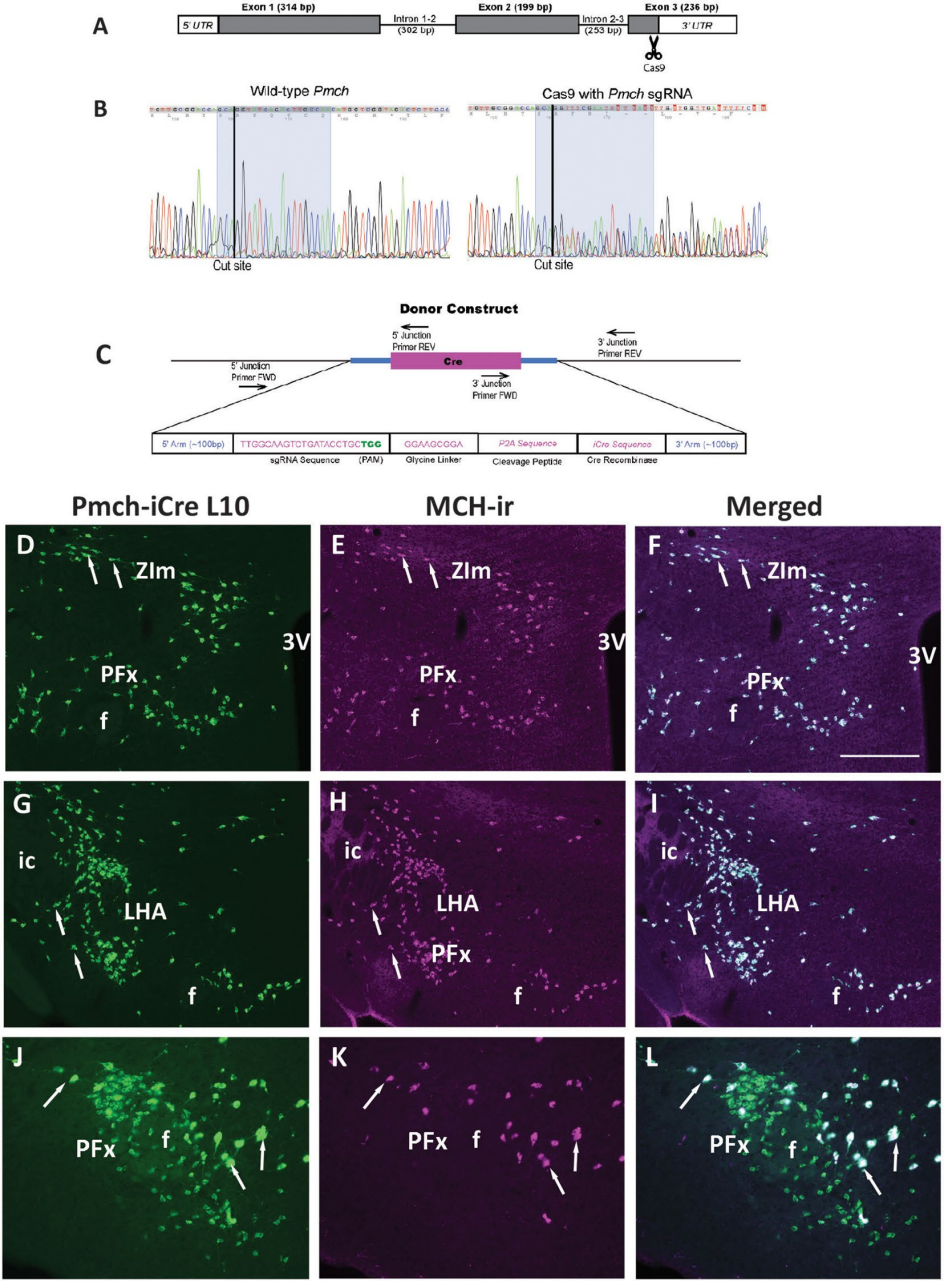
Generation of a *Pmch*-iCre knock-in mouse model. (**A**) Schematic illustration showing site in exon 3 of the murine *Pmch* gene where the construct containing the iCre sequence was inserted. (**B**) Sanger sequencing chromatogram showing site of iCre insertion in wild type (left panel) and in sgRNA efficiency tested (right panel) blastocysts. Note that DNA of wild type blastocyst shows single resolved peaks while DNA of tested blastocyst exhibits “peaks on peaks” indicative of correct site excision, non-homologous end joining and subsequent insertion/deletion (indel) mutations. (**C**) Schematic diagram of the donor construct used to generate *Pmch*-iCre mice showing the position of primers at 5’ and 3’ junction sites used to confirm integration of the donor construct in the correct orientation. (**D-L**) Fluorescent images showing the distribution of iCre-induced EGFP expression (**D**, **G**, **J**), MCH-immunoreactive (MCH-ir) neurons (**E**, **H**, **K**), and merged images (**F**, **I**, **L**) in three rostro-to-caudal hypothalamic levels. Note high colocalization rate in the medial zona incerta (ZIm), lateral hypothalamic area (LHA) and anterior perifornical area (PFx) and the high number of EGFP+/MCH- cells in the posterior PFx (PFx, image 73 of Allen Mouse Brain Atlas). Arrows indicate examples of neurons coexpressing both EGFP and MCH-ir. Abbreviations: 3 V, third ventricle; f, fornix; ic, internal capsule. Scale bar: D-I = 400 μm; J-L = 200 μm

CRISPR/Cas9 technology was used to insert codon-improved Cre recombinase (iCre) into the *Pmch* gene between the final protein-coding codon and the termination codon of the mouse *Pmch* (Ensembl gene ENSMUSG00000035383) in the same reading frame, as previously described [49, 50]. The endogenous *Pmch* polyadenylation signal in the 3’ UTR was kept intact (Fig. 1A).

The CRISPOR algorithm was used to identify specific single guide RNA targets (sgRNA) predicted to cut the chromosome near codon 165 [51]. The sgRNA C258G1 (Table 1) with a high cutting frequency determination (CFD) specificity score of 88 was selected [52, 53]. Recombinant *Streptomyces pyogenes* Cas9 endonuclease was obtained from MilliporeSigma (CAS9PROT). C258G1 was assembled into sgRNA/Cas9 ribonucleoprotein (RNP) complexes (30 ng/µL sgRNA + 50 ng/µL Cas9) and tested by mouse zygote microinjection to determine the efficiency in inducing chromosome breaks [54, 55]. Briefly, after RNP microinjection, zygotes were cultured in vitro to the blastocyst stage (about 64 cells per blastocyst). DNA was extracted from individual blastocysts and subjected to PCR and DNA sequencing to identify small insertions/deletions (indels) at the Cas9/sgRNA cut sites, indicative of non-homologous end joint (NHEJ) repair of double strand breaks (DSB) induced by sgRNA/Cas9 complexes (Fig. 1B, C). Primer sequences used for PCR amplification and Sanger sequencing are listed in Table 1.

**Table 1:** Primer sequences used in the generation and validation of the *Pmch*-iCre and *Pmch*^*ΔVglut2*^ mice

Target	Sequence	Purpose
sgRNA C258G1	TTGGCAAGTCTGATACCTGC (TGG)	Single-guide RNA for the generation of *Pmch*-iCre mice
Blastocyst DNA	F CCATAGGAAGGAGAGATTTTGACAGTGAG R GCAGAATTATGCAGAACTTTTGTGAGGTT	Amplify blastocyst DNA around cut site for sequencing
iCre Primer	F GACAGGCAGGCCTTCTCTGAA R CTTCTCCACACCAGCTGTGGA	Check for presence of iCre recombinase sequence
5’ Junction Site	F GAGATTTTGACATGCTCAGGTGT R CAGGTGCTGTTGGATGGTCT	Verify orientation of donor construct integration
3’ Junction Site	F GCCCTTCTGACTCCAATGCT R TGTGAGGTTTAATGCACACGTC	Verify orientation of donor construct integration
*Slc17a6* ISH (radioisotope)	F CCGGGGAAAGAGGGGATAAAG R GTAGACGGGCATGGATGTGA	Validation of Slc17a6-Exon2 BaseScope probe

Once effective Cas9 cleavage was verified, a Megamer^®^ single-stranded DNA donor (Integrated DNA Technologies) was synthesized to serve as a template for high-fidelity homologous directed repair (HDR) which included a P2A self-cleaving peptide sequence with a Gly-Ser-Gly linker between the last *Pmch* codon and the start of the iCre sequence and arms of homology (5’ end: 100 bp; 3’ end: 101 bp of genomic sequence) flanking the sgRNA target [56]. Silent base substitutions were made in these homologous arms to discourage repeated recognition and cleavage at the target site as is routine at the UM Transgenic Animal Core [54, 57].

Pronuclear microinjections were performed as previously described [58]. The microinjection mixture contained sgRNA C258G1 (30 ng/µL) complexed with WTCAS9 protein (50 ng/µL) and 10 ng/µL of ssDNA. Surviving zygotes were transferred to pseudopregnant females. One hundred and six potentially gene-edited generation zero (G0) founders were screened for the iCre knock-in by PCR amplicon sequencing. Those positive for iCre were subsequently sequenced at the 5’ and 3’ junctions to verify correct orientation of the donor fragment. Primer sequences used for genotyping are listed in Table 1. G0 founder animals carrying the mutation were mated to wild type mice to verify germline transmission, confirmed by genotyping of G1 offspring for iCre as described above.

Presence of functional iCre was confirmed by mating iCre positive G1 mice to B6;129S4-*Gt(ROSA)26Sor*^*tm9(EGFP/Rpl10a)Amc/*^J (LSL-EGFP-L10A) mice (JAX^®^ stock # 024750) [59, 60] and screening the brains of the resulting adult male and female G2 offspring for EGFP expression (*n* = 3/sex). In addition, due to the transitory expression of *Pmch* in lactating females [44, 61, 62] brains of *Pmch*-iCre; LSL-EGFP-L10A mice on day 19 of lactation (*n* = 3) were also evaluated.

### Validation of *Pmch*-iCre mice

Mice were deeply anesthetized with isoflurane and perfused with 10% normal buffered formalin. Brains were dissected, postfixed in the same fixative for 2 h, and cryoprotected overnight in 20% sucrose in 0.1 M PBS. Coronal Sections. (30-µm thickness, 4 series) were collected with a freezing microtome and stored in a cryoprotectant solution (20% glycerol/30% ethylene glycol in PBS) at − 20 °C. Dual-label immunohistochemistry (IHC) for MCH and EGFP was performed in one series of brain sections. Briefly, sections were incubated in 3% normal donkey serum (NDS) diluted in PBS + Triton-X100 (0.25%) for 30 min followed by overnight incubation in primary antisera chicken anti-EGFP (1:10,000, Aves Labs, AB_2307317) and rabbit anti-MCH (1:5,000, Phoenix Pharmaceuticals, AB_2722682). Sections were rinsed in PBS and incubated in secondary antisera (1:500 goat anti-chicken conjugated to Alexa Fluor™ 488, AB_2534096, and 1:500 donkey anti-rabbit conjugated to Alexa Fluor™ 594, AB_141637) for 1 h (Thermo Fisher Scientific). Sections were mounted onto gelatin-coated slides, air-dried, and cover slipped with Fluoromount G mounting medium (EMS).

### Quantification methods

Number of EGFP + and MCH immunoreactive (MCH-ir) neurons from male and female mice were quantified to define rate of colocalization (*n* = 3/sex). Number of MCH-ir neurons in *Pmch*-iCre EGFP male and female mice was also compared with C57BL6/J wildtype mice to assess potential changes in the expression of MCH immunoreactivity induced by insertion of iCre (*n* = 3/sex and genotype). Only one representative section from one side of each site (ZIm, LHA and PFx) was quantified. Brain levels were defined according to the Allen Mouse Brain Atlas.

### Generation and validation of mice lacking VGLUT2 (*Slc17a6* gene) in MCH neurons

Mice homozygous for *Pmch-*iCre were crossed with *Slc17a6*^*tm1Lowl*^/J (*Vglut2*^*flox*^) mice (JAX^®^ stock # 012898) [63, 64] to generate *Pmch-*iCre^+/-^; *Vglut2*^*fl/+*^ mice. *Pmch-*iCre^+/-^; *Vglut2*^*fl/+*^ mice were crossed to *Vglut2*^*flox*^ mice to generate *Pmch-*iCre^+/-^; *Vglut2*^*fl/fl*^ mice (*Pmch*^*ΔVglut2*^, conditional KO group) and *Pmch-*iCre^-/-^; *Vglut2*^*fl/fl*^ mice (*Vglut2*^*flox*^, control group).

For genotyping and validation of *Vglut2* exon-2 excision in *Pmch* neurons, we extracted DNA from fresh frozen samples of cerebellum, cerebral cortex and hypothalamus of *Vglut2*^*flox*^ and *Pmch*^ΔVglut2^ mice and performed PCR amplifications using the RED Extract-N-Amp Tissue PCR Kit #XNAT (SigmaAldrich) (*n* = 3 males/genotype). Outer primers flanking the *Vglut2* exon 2 were used [18] to detect a 536 bp DNA fragment indicating its excision.

To assess the extent of *Slc17a6* (*Vglut2*) deletion in MCH neurons we performed a chromogenic ISH using BaseScope Duplex (*n* = 3/sex and genotype). BaseScope was used due to the short probe sequence required to specifically target exon 2 of *Slc17a6* [64]. Fresh frozen 16-µm coronal sections were collected on Superfrost Excell slides (Fisher) and stored at − 80 °C. Tissue sections were fixed in 10% normal buffered formalin for 30 min and dehydrated in ethanol. Endogenous peroxidase was blocked with H_2_O_2_ for 10 min and tissue was digested with Protease IV for 10 min at room temperature. Sections were hybridized with BaseScope probes targeting *Pmch* (BA-Mm-Pmch-3zz-st-C1, # 890511-C1) or iCre (BA-iCre-3zz-st-C1, # 1254171-C1) and a custom-made probe to detect exon 2 of *Slc17a6* (BA-Mm-Slc17a6-1zz-st-C2, # 1240891-C2) for 2 h at 40 °C. Signal was detected with the BaseScope Duplex Detection Reagents ACD, #323810, using green (*Pmch* or iCre) and Fast RED (*Slc17a6*) and counterstained with 50% Gill’s hematoxylin for 30 s.

Single-labeled ISH using radiolabeled probes was performed in wild type mice as a control for the customized *Slc17a6*-exon2 BaseScope probe. Briefly, a series of brain sections were mounted onto SuperFrost plus slides (Thermo Fisher Scientific), fixed in 10% buffered formalin, dehydrated in ethanol and cleared in xylene, as previously described [44, 54, 64]. The *Slc17a6* DNA template was generated from mouse hypothalamic RNA by PCR amplification (*n* = 3, C57BL6/J males). Primers used to amplify a 909 base-pair sequence in the gene encoding the *Slc17a6* gene are described in Table 1. The antisense radiolabeled *Slc17a6* ^35^S riboprobe was generated by in vitro transcription [35]. The 35S-labeled riboprobe was diluted in hybridization solution (50% formamide, 10 mM Tris–HCl pH 8.0, 5 mg tRNA, 10 mM dithiothreitol, 10% dextran sulfate, 0.3 M NaCl, 1 mM EDTA, and 1× Denhardt’s solution), and brain slices were hybridized overnight at 57 °C. Slides were incubated in 0.002% RNase A followed by stringency washes in sodium chloride-sodium citrate buffer (SSC). Sections were dipped in NTB autoradiographic emulsion (Kodak) and stored in light-protected slide boxes at 4 °C for 2 weeks. Signal was developed with developer and fixer (Carestream, Rochester, NY, USA), and slides were cover slipped with DPX (EMS) mounting medium.

### Reproductive phenotyping

Starting at weaning, on postnatal day 21 (PD21), males and females were monitored for external indices of puberty onset and completion. Males were monitored for balanopreputial separation (BPS), defined as the day the prepuce separates fully from the glans, and females were assessed for vaginal opening (VO) [65, 66]. Once females exhibited VO, vaginal lavage was performed daily in the morning, from 9:00 to 11:00AM. Vaginal cytology was assessed to determine the day of first estrus and, subsequently, estrous cyclicity [67]. Because body weight can cause indirect effects on pubertal timing and estrous cyclicity, mice were weighed daily up to postnatal day 60 (P60) to assess body weight at the day of BPS, VO and first estrus. Estrous cycles were monitored for 40 days, starting on P75. Three cohorts of mice were evaluated (*n* = 6–11/genotype in each cohort). One cohort of mice on HFD (*n* = 5–6/genotype) was also evaluated for time of VO, first estrus and cyclicity (25 days).

Fertility of mature female mice was further evaluated by measuring latency to pregnancy. Female *Vglut2*^*flox*^ and *Pmch*^*ΔVglut2*^ littermates were housed two to a cage such that each cage contained a control (*Vglut2*^*flox*^) and a conditional knockout (*Pmch*^*ΔVglut2*^) mouse. A sexually experienced wild-type male mouse was assigned to each cage. *Pmch*^*ΔVglut2*^ males were housed with a *Vglut2*^*flox*^ female to assess fertility of mature males. Latency to pregnancy, litter size and offspring weight was recorded at weaning day (P21). Two cohorts of mice were evaluated for fertility (*n* = 5–8/genotype in each cohort).

### Metabolic phenotyping

Two cohorts of *Vglut2*^*flo*x^ and *Pmch*^*ΔVglut2*^ mice (*n* = 6–11/genotype in each cohort) maintained on regular chow diet (3 kcal/g) were weighed weekly starting at P60 (~ 8 weeks of age) until P135-140 (weeks 19–20) and before euthanasia at 24 weeks of age. At 19–20 weeks of age, they were subjected to comprehensive metabolic phenotyping carried out in the University of Michigan Mouse Metabolic Phenotyping Center (MMPC-Live, mmpc.med.umich.edu). Male and female mice were single housed on a 12:12 light/dark cycle (lights on at 6:00AM) and transferred to MMPC-Live one week before phenotyping. Mice were then transferred to the Promethion (SABLE system) cages for measurement of food intake, locomotor activity, respiratory exchange ratio, glucose and fat oxidation and energy expenditure for three consecutive days [48, 68, 69]. The environment was kept at room temperature (20–23 °C). Body composition was measured using an EchoMRI, 4in1-500.

Two additional cohorts of *Vglut2*^*flox*^ and *Pmch*^*ΔVglut2*^ males (total *n* = 9–11/genotype) and females (total *n* = 9–12/genotype) were fed a HFD (HFD, Teklad TD88137, 42% calories from fat, 4.5 kcal/g) for 12 weeks beginning at 7 weeks of age. Mice were weighed weekly and sent to MMPC-Live for comprehensive metabolic phenotyping at 19–20 weeks of age and were weighed before euthanasia at 24 weeks of age.

For assessment of glucose homeostasis, mice were fasted overnight to determine fasting glucose levels (also performed in the MMPC-Live with the same cohorts used for metabolic phenotyping). Glucose (25%) was given at 9:00 AM via oral gavage at 2 g/kg. Blood samples were collected prior to (time 0) and after administration at 15, 30, 60, and 120 min via tail vein. Blood levels of glucose were measured using a glucometer (Acucheck, Roche) and plasma levels of insulin were determined using a rat/mouse insulin ELISA kit (Millipore). The total area under the curve (AUC) for glucose and insulin were calculated using the trapezoidal rule [70]. Homeostatic model assessment of insulin resistance index used as a surrogate measure for potential changes in insulin resistance. It was calculated as the product of the fasting insulin in µU/mL × fasting glucose in mg/dL divided by 405 (constant that adjust units of measurement into an index) [71]. Glucose tolerance test (GTT) and insulin assay were performed at 20–21 weeks of age (after metabolic phenotyping).

### Imaging and data analysis

Slides were examined under an Axio Imager M2 Microscope or a SteREO DiscoveryV8 (Zeiss). The digital Allen Mouse Brain Atlas was used as a reference to determine relative location within the hypothalamus and identify the primary sites containing MCH and EGFP immunoreactivity. Images were acquired with a digital camera (Axiocam, Zeiss) using Zen software. For data illustration, only sharpness, contrast, and brightness were adjusted. Box and whisker plots were used to show variability within a genotype.

Data are expressed as mean ± SEM. The unpaired two-tailed Student t-test with Welch’s correction, multiple unpaired t-test with false discovery rate (FDR, q-value), one- and two-way ANOVA and Tukey multiple comparisons or Sidáák corrections were performed using Graph Pad Prism software v. 10.2.2. Analysis of covariance (ANCOVA) were performed using CalRapp.org (web application for indirect calorimetry analysis). [72] A p-value of< 0.05 was considered significant in all analyses. Detail of the statistical analysis are described in the corresponding results section or figure legend.

## Results

### Production and validation of *Pmch*-iCre mouse model

The functionality of the iCre recombinase was confirmed by mating G1 *Pmch*-iCre mice to a Cre-inducible reporter line (LSL-EGFP-L10A) and by checking the brains of adult male and female G2 offspring for appropriate EGFP expression using immunohistochemistry for MCH and EGFP. Above 98% of all MCH-ir neurons of different sites and sexes coexpressed EGFP (Table 2). About 95% of *Pmch*-iCre EGFP neurons in the ZIm/IHy and LHA, and 85% in the anterior PFx were found to express MCH-immunoreactivity in both sexes (Table 2 and Fig. 1D-I). A dense cluster of very small and round EGFP+/MCH- cells were observed surrounding the fornix, particularly at the level of the posterior aspect of the tuberal LHA, similar to what we reported in the BAC transgenic line [44] (Fig. 1J-L). At that level, MCH-ir was detected in 35–40% of EGFP + cells (Table 2). No difference in the colocalization rate was observed comparing sexes.

**Table 2:** Coexpression of EGFP and MCH immunoreactivity in *Pmch*-iCre EGFP mice of both sexes. Differences between sexes were evaluated using multiple *t* test (*n* = 3/sex and area, df = 4). Abbreviations: IHy, incertohypothalamic area; LHA, lateral hypothalamic area; PFx, Perifornical area; ZIm, Rostromedial Zona incerta; Atlas images according to the Allen mouse brain atlas.

Hypothalamus	Atlas	Male (%)	Female (%)	*p* value	t ratio
Areas	Images	EGFP + MCH/ **total MCH**	EGFP + MCH/ **total MCH**	Male vs. Female	Male vs. Female
ZIm (IHy)	69	97.74 ± 1.18	100 ± 0	0.13	1.90
LHA	71	98.74 ± 0.63	98.65 ± 0.74	0.93	0.09
Anterior PFx	69	99.50 ± 0.49	97.52 ± 1.30	0.22	1.43
Posterior PFx	73	100 ± 0	99.51 ± 0.48	0.37	1.00
**Areas**	**Images**	**EGFP + MCH/** **total EGFP**	**EGFP + MCH/** **total EGFP**	**Male vs. Female**	**Male vs. Female**
ZIm (IHy)	69	96.82 ± 3.17	100 ± 0	0.37	1.00
LHA	71	94.42 ± 2.79	95.42 ± 3.86	0.84	0.211
Anterior PFx	69	81.43 ± 9.09	85.55 ± 5.57	0.72	0.38
Posterior PFx	73	35.48 ± 7.10	40.63 ± 8.67	0.67	0.46

**Fig. 2:**
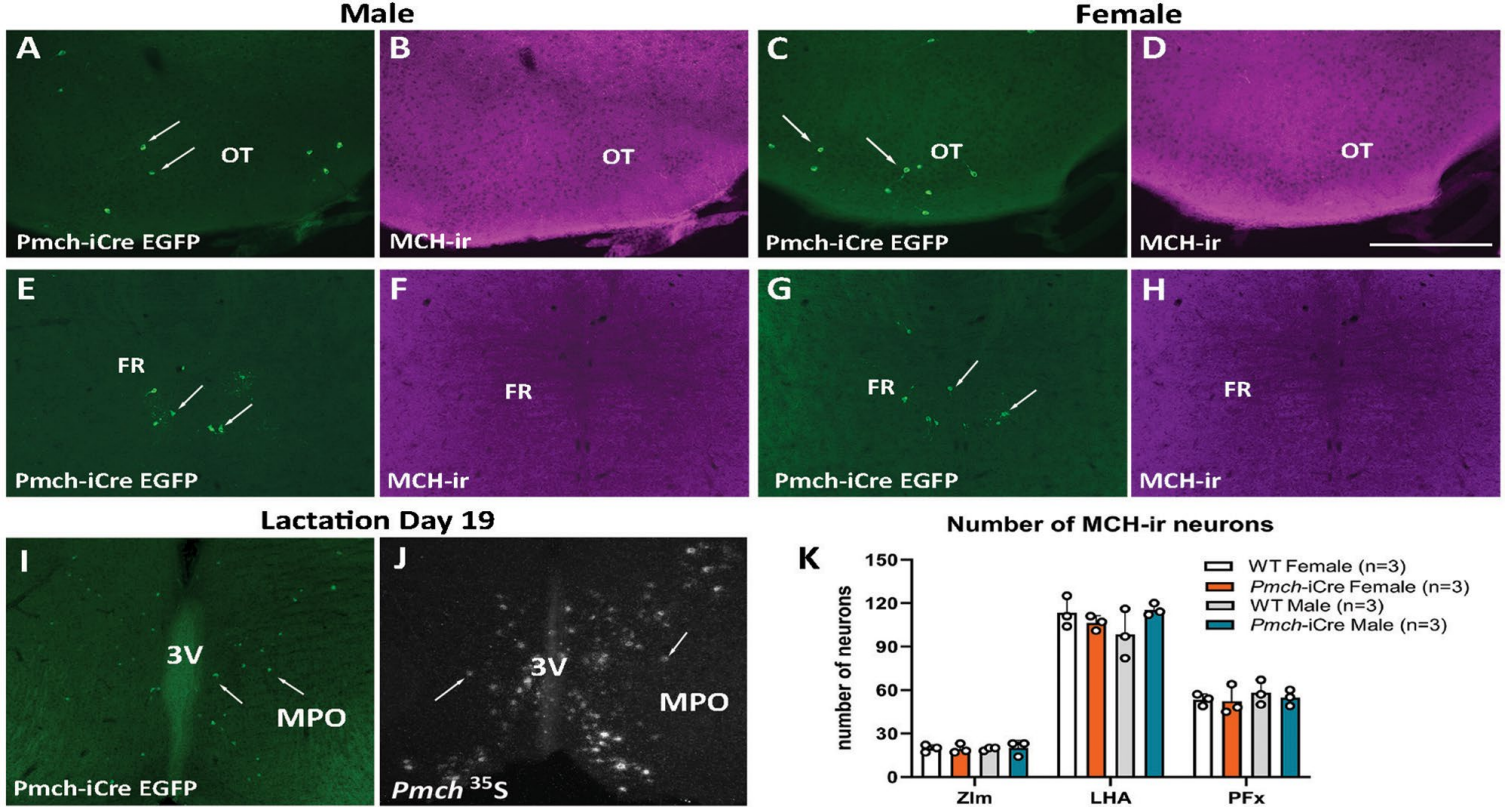
Additional sites of *Pmch*-iCre EGFP expression in the mouse brain. (**A**-**H**) Fluorescent images showing *Pmch*-iCre EGFP (arrows) and lack of MCH immunoreactive (MCH-ir) neurons in the olfactory tubercle (OT, A-D) and in the pontine reticular nucleus (PRN, **E**-**H**). (**I**) Fluorescent image showing *Pmch*-iCre EGFP neurons (arrows) in the preoptic area of a late-lactation (day 19) dam. (**J**) Dark field image showing distribution of *Pmch* mRNA (arrows, hybridization signal) in the preoptic area of a late-lactation dam. (**K**) Bar graph showing number of MCH-ir neurons in the ZIm, LHA, and PFx of *Pmch*-iCre EGFP vs. C57BL6/J wildtype male and female mice. Note that the insertion of iCre did not change the number of MCH-ir neurons in either sex or area evaluated. Two-way ANOVA, Tukey’s multiple comparison test, number of MCH-ir neurons df = 24 (*n* = 3/sex and genotype) in the ZIm *p* > 0.98, in the LHA *p* > 0.10, in the PFx *p* > 0.80. Abbreviations: 3 V, third ventricle; DR, dorsal raphe; f, fornix; MPO, medial preoptic nucleus. Scale bars: A-J = 200 μm

EGFP-ir, but not MCH-ir, was also detected in brain sites previously described to show *Pmch* mRNA in rats [73] i.e., olfactory tubercle and pontine reticular formation in both sexes (Fig. 2A-H), and in a few brainstem nuclei. Further studies are needed to determine whether these findings are a result of *Pmch* expression during development.

EGFP-ir was also observed in the POA and PVH of dams on lactation day 19 in accordance with previously documented *Pmch* mRNA expression in late lactation in mice and rats [44, 61, 62, 74] (Fig. 2I-J). To verify that iCre knock-in did not alter the *Pmch* gene itself, we compared the number of MCH-ir neurons in male and female *Pmch*-iCre and wild type C57BL/6 mice and found no difference between sexes or genotypes (Fig. 2K).

### Production and validation of mice with deletion of *Vglut2* in MCH neurons (*Pmch*^*ΔVglut2*^)

The *Pmch-*iCre mouse line was used to investigate the reproductive and metabolic phenotyping of male and female mice that lack glutamatergic neurotransmission in MCH neurons (*Pmch*^*ΔVglut2*^ mice). Cre-negative littermates homozygous for the floxed allele (*Vglut2*^*flox*^ mice) were used as controls.

**Fig. 3:**
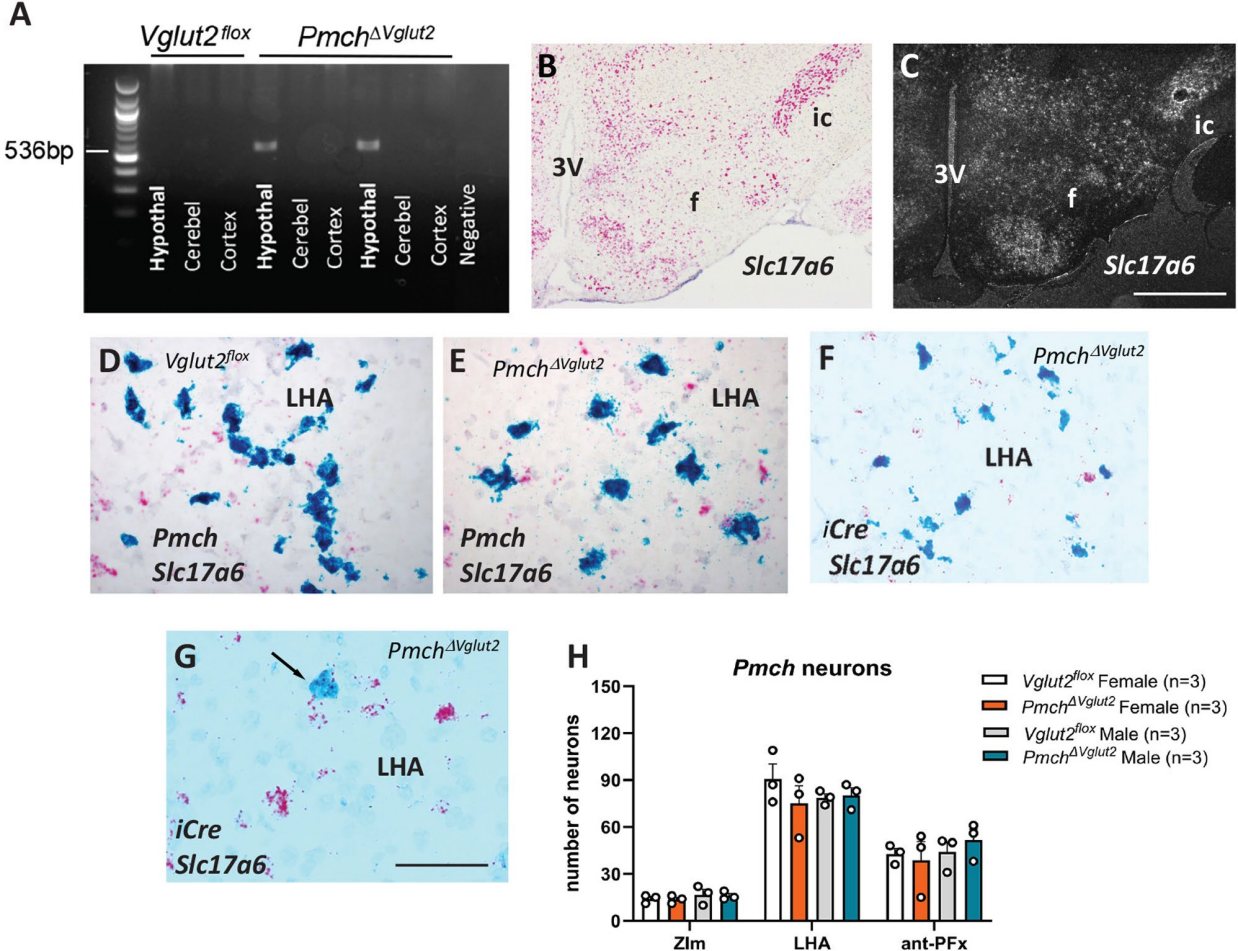
Validation of deletion of *Vglut2* (*Slc17a6* gene) in mouse MCH neurons. (**A**) Dark field image of gel electrophoresis showing iCre excision of *Vglut2*-exon2 obtained with PCR. Note that only hypothalamic samples *Pmch*_*ΔVglut2*_ mice show 536 bp amplicon. (**B**) Bright field image showing distribution of *Vglut2* revealed by in situ hybridization using a customized probe specifically targeting exon 2 of *Vglut2* gene. (**C**) Dark field image showing distribution of *Vglut2* revealed by a probe labeled with radioisotope ( [35]S) targeting 909 base pairs the *Vglut2* gene. (**D-E**) Bright field imaging showing *Pmch* (blue) and *Vglut2*-exon 2 (magenta) expression in *Vglut2*^*flox*^ and *Pmch*_*ΔVglut2*_ mice using BaseScope technology. (**F**) Bright field image showing lack of coexpression of *Vglut2* in iCre neurons of a *Pmch*_*ΔVglut2*_ mouse. (**G**) Bright field image showing coexpression of *Vglut2* in iCre neurons (arrow). Coexpression of observed in about 5% of iCre neurons in the LHA. (H) Bar graph showing that the number of *Pmch* neurons remains intact following deletion of *Vglut2* in male and female *Pmch*_*ΔVglut2*_ mice. Two-way ANOVA Tukey test *p* > 0.98 for ZIm, *p* > 0.35 for LHA and *p* > 0.051 for PFx, df = 24 comparing genotypes and sexes. Abbreviations: 3 V, third ventricle; f, fornix; ic, internal capsule; LHA, lateral hypothalamic area. Scale bar: B-C = 500 μm; D-G = 100 μm

As an initial step for validation of iCre efficiency, we assessed DNA recombination in the hypothalamus and used samples of cerebellum and cerebral cortex as control. We found the 536 bp fragment representing the excision of *Vglut2 exon 2* only in the hypothalamus of the *Pmch*^*ΔVglut2*^ mice (Fig. 3A). To determine the extension of deletion, we performed dual labeling fluorescent in situ hybridization but found that fluorescent probes lacked adequate specificity to label exon 2 of *Vglut2* due to its small size. The validation of the conditional deletion was therefore performed using the BaseScope assay and a customized probe specifically targeting the exon 2 of *Slc17a6* gene that is flanked by loxP sites in the *Vglut2*^*flox*^ mice. The custom-made BaseScope probe has been documented [64] and was further corroborated by comparing the pattern of distribution of *Vglut2* and *Vglut2* exon 2 hybridization signal (Fig. 3B-C). However, due to the dense *Pmch* expression, successful deletion of *Vglut2* was difficult to ascertain (Fig. 3D-E). We therefore used the BaseScope iCre probe and found deletion of *Vglut2* in 93 ± 0.65% of ZIm *Pmch*-iCre neurons, 95.45 ± 0.95% of LHA *Pmch*-iCre neurons and in 91.1 ± 0.6% of PFx *Pmch*-iCre neurons (Fig. 3F-G). Due to lack of sex differences in iCre-induced EGFP, males were used for the recombination test and females were used for ISH. We further assessed if lack of *Vglut2* has any effect in the number of *Pmch* neurons in distinct hypothalamic sites and found no difference in either ZIm, LHA or PFx of male and female *Pmch*^*ΔVglut2*^ vs. *Vglut2*^*flox*^ mice (Fig. 3H).

### Female *Pmch*^*ΔVglut2*^ mice exhibit delayed puberty and increased latency to pregnancy

**Fig. 4:**
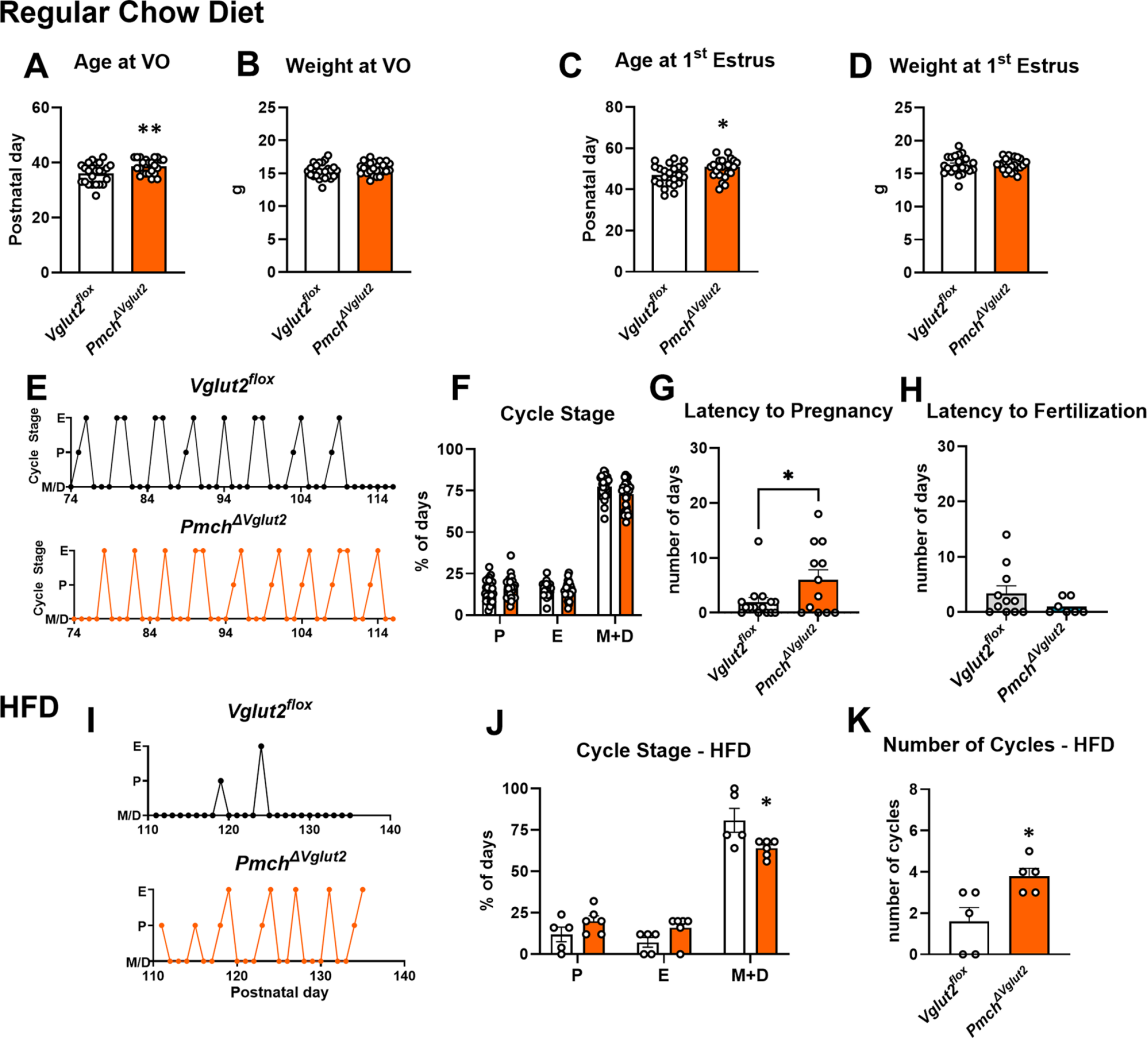
Female *Pmch*^*ΔVglut2*^ mice exhibit delayed puberty, increased latency to pregnancy and regular cyclicity on HFD. (**A**-**D**) Age at vaginal opening (VO) and first estrus, as well as body weight in both developmental stages. *Pmch*_*ΔVglut2*_ mice show delayed puberty onset and completion but no change in body weight (*n* = 25 *Vglut2*^*flox*^, *n* = 24 *Pmch*_*ΔVglut2*_). Two-tailed t-test, Welsh’s correction. (**A**) *p* = 0.0083, F (*p* = 0.18), t = 2.767, df = 42.59. (**B**) *p* = 0.22, F (*p* = 0.44), t = 1.240, df = 44.37. (**C**) *p* = 0.025, F (*p* = 0.66), t = 2.31, df = 43.62. (**D**) *p* = 0.96, F (*p* = 0.09), t = 0.040, df = 39.15. (**E**) Example of estrous cycles (**F**) Percentage of days in each estrous stage (P, proestrus; E, estrus; M, metestrus; D, diestrus; *n* = 25 *Vglut2*^*flox*^, *n* = 24 *Pmch*_*ΔVglut2*_). Two-way ANOVA Sidak’s test: Interaction *p* = 0.061, F = 2.84; row (estrous stage) *p* < 0.0001, F = 1738; column (genotype) *p* = 0.174, F = 1.87. (**G**) Latency to pregnancy in days (*n* = 15 *Vglut2*^*flox*^, *n* = 12 *Pmch*_*ΔVglut2*_). Two-tailed t-test *p* = 0.038, F (*p* = 0.02), t = 2.17, df = 25. (**H**) Latency to fertilize a female (*n* = 11 *Vglut2*^*flox*^, *n* = 7 *Pmch*_*ΔVglut2*_). Unpaired two-tailed t-test, *p* = 0.20, t = 1.323, df = 16. (**I**) Example of estrous cycles of mice on high fat diet (HFD). (**J**) Percentage of days in each estrous stage of mice on HFD (*n* = 5 *Vglut2*^*flox*^, *n* = 6 *Pmch*_*ΔVglut2*_). Two-way ANOVA Sidak’s test: interaction *p* = 0.0043, F = 6.69; row (estrous stage) *p* < 0.0001 F = 145.3; column (genotype) diestrus *p* = 0.017. (**J**) Number of cycles of mice on HFD. Two-tailed t-test *p* = 0.022, F (*p* = 0.27), t = 2.84, df = 8. *Vglut2*^*flox*^ mice in white and *Pmch*_*ΔVglut2*_ in orange

Male and female mice were monitored for external signs of pubertal development starting at weaning (P21). No differences were observed in day of BPS (*Vglut2*^*flox*^: 30.84 ± 0.64 days of age, *Pmch* ^*ΔVglut2*^: 30.67 ± 0.57 days of age) or weight at day of BPS (*Vglut2*^*flox*^: 15.86 ± 1.33 g, *Pmch*^*ΔVglut2*^ 16.24 ± 0.33 g, respectively) between *Pmch*^*ΔVglut2*^ and *Vglut2*^*flox*^ mice. However, female *Pmch*^*ΔVglut2*^ exhibited a delay for vaginal opening (puberty onset) and first estrus (puberty completion), with no difference in body weight at either time point (Fig. 4A-D).

Total time spent in each estrous cycle stage (proestrus, estrus, and metestrus/diestrus) as well as average cycle length were consistent between *Vglut2*^*flox*^ and *Pmch*^*ΔVglut2*^ females (Fig. 4E-F). *Pmch*^*ΔVglut2*^ male and female mice were fertile. No difference in average litter size (8.0 ± 2.5 vs. 7.3 ± 2.5 pups in *Vglut2*^*flox*^) or offspring weight at weaning (7.9 ± 2.2 g vs. 8.3 ± 1.4 in *Vglut2*^*flox*^) was observed. Female *Pmch*^*ΔVglut2*^ mice however showed increased latency to pregnancy (*p* = 0.02, Fig. 4G). No difference in breeding latency to fertility (Fig. 4H) or litter size (7.75 ± 0.43 vs. 7.98 ± 0.62 pups in *Vglut2*^*flox*^ *n* = 5–7, *p* = 0.79) was observed in males.

Because HFD disrupts the estrous cycle in mice and rats [75, 76] we assessed whether lack of glutamatergic neurotransmission in MCH neurons attenuates this effect. Notably, *Pmch*^*ΔVglut2*^ females on HFD showed regular estrous cycles, percentage of days in each cycle stage and estrous cycle length compared to *Vglut2*^*flox*^ mice (Fig. 4I-K).

### *Pmch*^*ΔVglut2*^ female mice on regular chow diet exhibit late onset leanness

The metabolic effects of conditional deletion of VGLUT2 in MCH neurons have been documented in male mice [16, 18] but whether females are differentially affected is unknown. We performed comprehensive metabolic phenotyping in both sexes under distinct feeding regimens (i.e., regular chow and high-fat diet). Data from different cohorts for each sex was pooled as differences were not observed between cohorts.

**Fig. 5:**
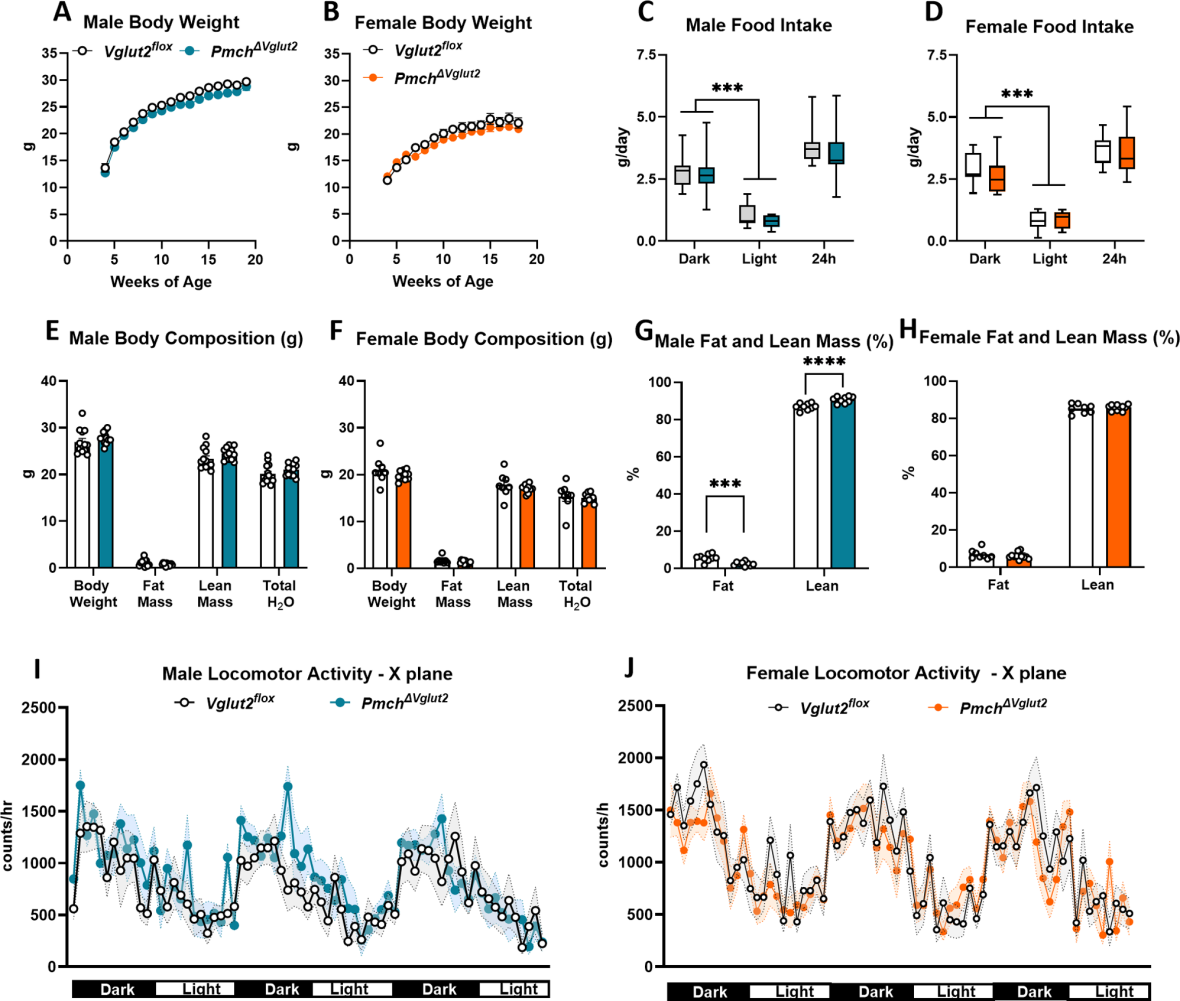
*Pmch*^*ΔVglut2*^ mice on regular chow diet exhibit late onset leanness. (**A**-**B**) Body weight trajectory of males (A, *n* = 16 *Vglut2*^*flox*^ *n* = 18 *Pmch*_*ΔVglut2*_) and females (B, *n* = 19/genotype) from 5–20 weeks of age. Two-way ANOVA repeated measures Sidák’s test *p* > 0.9 (males) and *p* > 0.20 (females). (**C-D**) Average food intake of males (**C**) and females (**D**) during dark and light phases, and 24 h. Two-way ANOVA, Tukey’s test. In C, interaction *p* = 0.70 F (2, 69) = 0.34, row (period) *p* < 0.001 F = 102.7, column (genotypes) *p* = 0.25, F = 1.35. In D, interaction *p* = 0.82 F = 0.19, row (period) *p* < 0.0001 F (2, 51) = 94.69, column (genotypes) *p* = 0.62, F = 0.24. (**E-H**) Total (**g**) and relative (%) fat and lean mass of males and females. Note a decrease in fat mass and increase in lean mass of *Pmch*_*ΔVglut2*_ males at 20 weeks of age. Two-way ANOVA Sidák’s test. In E, row factor (component) *p* < 0.001, column factor (genotype) *p* = 0.24, interaction *p* = 0.70. In F, row factor (component) *p* < 0.001, column factor (genotype) *p* = 0.74, interaction *p* < 0.001, df = 62. In G, fat mass *p* = 0.0005 t = 0.088 df = 32, and lean mass *p* < 0.0001 t = 4.7 df = 32. In H, fat mass *p* = 0.60 t = 0.90 df = 34, and lean mass *p* < 0.70 t = 0.74 df = 34. (**I-J**) Graphs showing locomotor activity (X-plane) of *Vglut2*^*flox*^ and *Pmch*_*ΔVglut2*_ males (**I**) and females (**J**) across 3 days. C-J performed in the MMPC in two trials. Cohorts were combined (total *n* = 7–9/sex and genotype). *Vglut2*^*flox*^ mice in white and *Pmch*_*ΔVglut2*_in blue (male) or orange (female)

On regular chow diet (3 kcal/g), neither male nor female *Pmch*^*ΔVglut2*^ mice showed differences in body weight, food intake, or absolute lean and fat masses compared to control (*Vglut2*^*flox*^) littermates at 20 weeks of age (Fig. 5A-F). Despite exhibiting no differences in body weight, *Pmch*^*ΔVglut2*^ male, but not female, mice had altered body composition relative to controls, with a reduced fat mass and increased lean mass (Fig. 5G-H, Supplementary Figure A). By 24 weeks of age, *Pmch*^*ΔVglut2*^ females, but not males, exhibited lower body weight than controls (21.60 ± 0.77 vs. 19.40 ± 0.38 g, *p* = 0.013, unpaired two-tailed *t*-test, t = 2.76, df = 17). Locomotor activity in all three planes was similar between genotypes in both sexes during the dark and light phases individually as well as across a 24 h period (Fig. 5I-J, X-plane *p* = 0.61, Y-plane *p* = 0.45 and Z-plane *p* = 0.96 for females, and X-plane *p* = 0.99, Y-plane *p* = 0.88 and Z-plane *p* = 0.99 for males, two-way ANOVA and Sidak’s multiple comparisons test). Fat and glucose oxidation were also unchanged by *Vglut2* deletion in both sexes during the dark and light phases individually and across a 24 h period (Tables 3 and 4).

**Table 3:** Average fat oxidation of both sexes on regular chow and HFD (*n* = 7–11/sex, genotype and diet). Average was calculated using the last two days in the SABLE system. Data was analyzed by two-way ANOVA and Tukey multiple comparison test. Data is represented as mean g per kg of lean body mass per hour in 24 h, dark and light phase ± SEM. No difference considering genotype, sex and period analyzed was observed

Sex and Diet	Period	*Vglut2* ^*flox*^	*Pmch* ^*ΔVglut2*^	*p* value
Female Regular Chow (3.0 kcal/g)	24 h	0.497 ± 0.1233	0.566 ± 0.0746	0.95
	Dark phase	0.253 ± 0.1702	0.266 ± 0.1118	0.99
	Light phase	0.777 ± 0.0895	0.906 ± 0.0495	0.76
Male Regular Chow (3.0 kcal/g)	24 h	0.258 ± 0.0327	0.275 ± 0.0528	0.99
	Dark phase	0.077 ± 0.0693	0.077 ± 0.0693	0.99
	Light phase	0.462 ± 0.0373	0.519 ± 0.0625	0.93
Female High Fat (4.5 kcal/g)	24 h	1.370 ± 0.1105	1.510 ± 0.0825	0.78
	Dark phase	1.465 ± 0.1112	1.541 ± 0.0896	0.95
	Light phase	1.269 ± 0.1162	1.474 ± 0.0824	0.51
Male High Fat (4.5 kcal/g)	24 h	1.118 ± 0.0709	1.103 ± 0.0954	0.99
	Dark phase	1.211 ± 0.0748	1.151 ± 0.0874	0.94
	Light phase	1.017 ± 0.0731	1.052 ± 0.1067	0.98

**Table 4:** Average glucose oxidation of both sexes on regular chow and high fat diets (*n* = 7–11/sex, genotype and diet). Average was calculated using the last two days in the SABLE system. Data was analyzed by two-way ANOVA with Tukey post-hoc test for multiple comparisons test. Data is represented as mean g per kg of lean body mass per hour in 24 h, dark and light phase ± SEM. No difference considering genotype, sex and period analyzed was observed

Sex and Diet	Period	*Vglut2* ^*flox*^	*Pmch* ^*ΔVglut2*^	*p* value
**Female ** **Regular Chow ** **(3.0 kcal/g)**	24 h	4.618 ± 0.2288	4.977 ± 0.1679	0.66
	Dark phase	5.851 ± 0.2789	6.449 ± 0.2818	0.24
	Light phase	3.228 ± 0.2427	3.332 ± 1330	0.98
**Male ** **Regular Chow ** **(3.0 kcal/g)**	24 h	4.605 ± 0.1395	4.480 ± 0.1067	0.93
	Dark phase	5.510 ± 0.2025	5.532 ± 0.1552	0.99
	Light phase	3.592 ± 0.2126	3.297 ± 0.1346	0.50
**Female ** **High Fat** **(4.5 kcal/g)**	24 h	2.454 ± 0.3023	2.639 ± 0.1309	0.94
	Dark phase	2.499 ± 0.3033	3.184 ± 0.1854	0.18
	Light phase	2.392 ± 0.3186	2.038 ± 0.1195	0.71
**Male ** **High Fat** **(4.5 kcal/g)**	24 h	2.510 ± 0.1455	2.323 ± 0.1280	0.86
	Dark phase	2.634 ± 0.1683	2.605 ± 0.1886	0.99
	Light phase	2.367 ± 0.1733	1.989 ± 0.1091	0.42

### *Pmch*^*ΔVglut2*^ mice on a HFD show no detectable disruption of daily feeding pattern

**Fig. 6:**
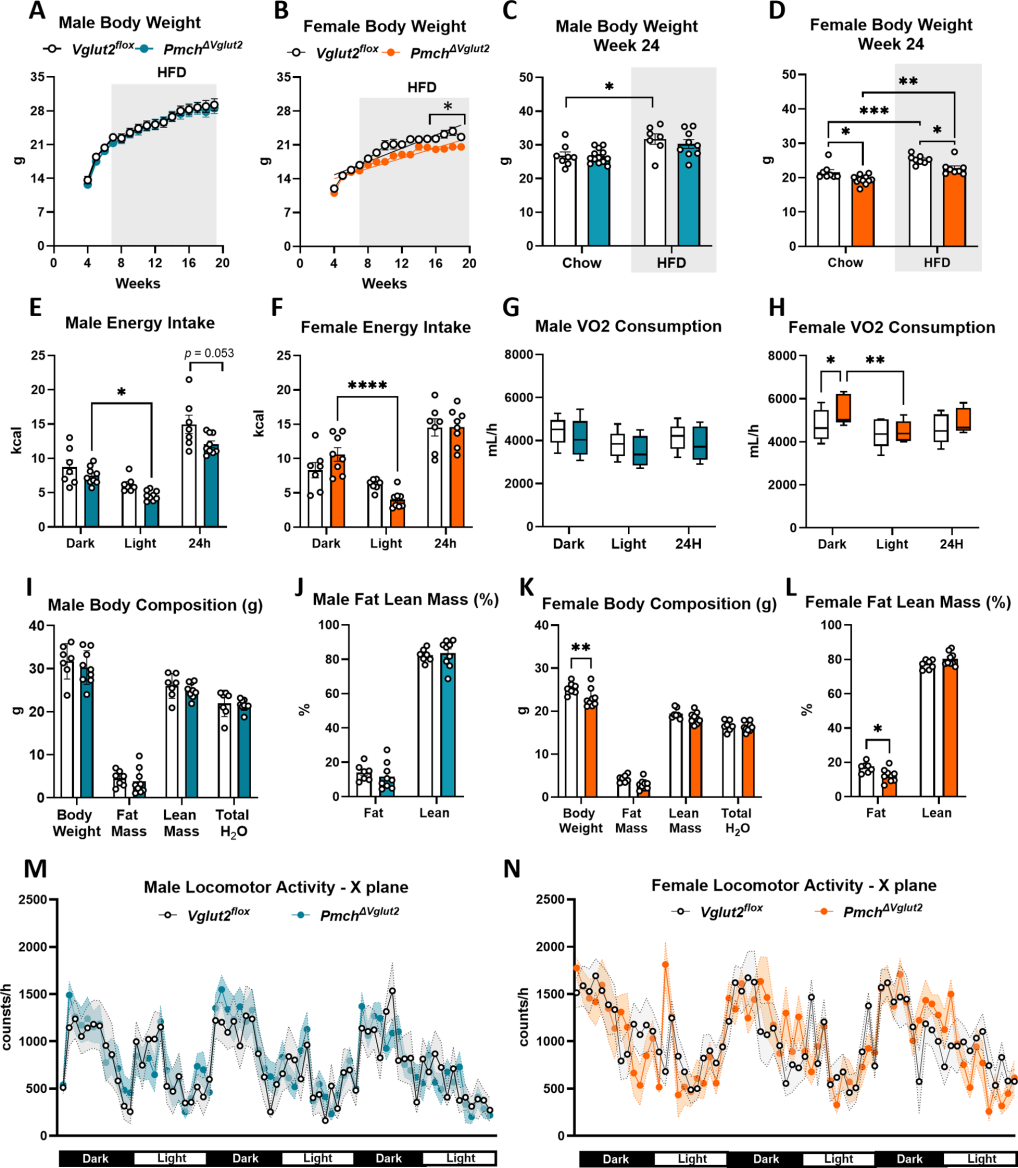
*Pmch*^*ΔVglut2*^ females gain less fat, and both sexes exhibit undetectable disruption of daily feeding pattern on HFD. (**A**-**B**) Body weight trajectory of *Vglut2*^*flox*^ and *Pmch*_*ΔVglut2*_males (A, *n* = 7–9/genotype) and females (B, *n* = 8/ genotype) on HFD. *Pmch*_*ΔVglut2*_females gain less weight compared to *Vglut2*^*flox*^ littermates. (A) Two-way ANOVA repeated measures, row (days) *p* < 0.001, column (genotype) *p* = 0.19, interaction *p* = 0.99. (**B**) Two-way ANOVA repeated measures, row (days) *p* < 0.0001, column (genotype) *p* = 0.008, interaction *p* < 0.0001 (**C-D**) Weigh gain of males (**C**) and females (**D**) mice comparing regular chow and HFD at 24 weeks of age. *Pmch*_*ΔVglut2*_females gain less weight in both diets. In C, * *p* = 0.030 df = 33 and in D, * *p* < 0.05, ** *p* = 0.0012, *** *p* = 0.0008, df = 31. (**E-F**) Average energy intake of males (**E**) and females (**F**). Note that whereas *Vglut2*^*flox*^ mice exhibited HFD-induced disruption daily feeding, *Pmch*^*ΔVglut2*^ mice maintained the daily pattern of food consumption. In E, * *p* = 0.035 df = 42 and in F, **** *p* < 0.0001 df = 39. (**G**) VO2 consumption in males. (**H**) VO2 consumption in females. * *p* = 0.04, ** *p* = 0.004 df = 42. (**I-L**) Body composition and relative (%) of fat and lean masses in males (I-J) and females (K-L). In K ** *p* = 0.0056 df = 56, and in L * *p* = 0.026 df = 28. (**M-N**) Locomotor activity across three days. C-L, two-way ANOVA and Tukey’s tests. Analysis performed in the MMPC in two trials with *n* = 4–5/sex and genotype every trial. Cohorts were combined for analysis (total *n* = 7–9/sex and genotype). *Vglut2*^*flox*^ mice in white and *Pmch*^*ΔVglut2*^in blue (**M**) or orange (**F**)

To determine whether latent metabolic changes might be revealed in mice under conditions of energy excess, two cohorts of male and female mice were fed a HFD (42% calories from fat, 4.5 kcal/g). After 12 weeks on HFD, female, but not male, *Pmch*^*ΔVglut2*^ mice had gained less weight compared to controls (Fig. 6A-D, Supplementary Figure B). At 24 weeks of age, body weight of males from both genotypes remained similar (*p* = 0.81), but whereas control mice exhibited increased weight gain on HFD (*p* = 0.03), *Pmch*^*ΔVglut2*^ male mice showed only a weak trend towards increased body weight (*p* = 0.063) compared to mice in regular chow diet (Fig. 6C-D). Total daily energy intake was not different between genotypes in either sex, although males showed a trend towards reduced daily (24 h) energy intake (*p* = 0.053). In contrast to control mice, which exhibited the expected disruption in daily feeding pattern [77]both male and female *Pmch*^*ΔVglut2*^ mice on a HFD showed no detectable disruption in daily pattern of food consumption, i.e., food intake remained higher in the dark phase (Fig. 6E-F).

*Pmch*^*ΔVglut2*^ and *Vglut2*^*flox*^ males on HFD were indistinguishable in oxygen consumption and energy expenditure (*p* = 0.57, Fig. 6G). *Pmch*^*ΔVglut2*^ female mice on HFD exhibited higher oxygen consumption in the dark phase (Fig. 6H, Supplementary Figure C) but no difference in a 24 h period compared to controls. Due to body mass differences, we used weight as a covariate and found no difference in either oxygen consumption (*p* = 0.75) or energy expenditure (*p* = 0.69). The difference in oxygen consumption during the dark phase is likely due to the disrupted daily pattern of food consumption observed in *Vglut2*^*flox*^ mice on HFD, an effect that was prevented in *Pmch*^*ΔVglut2*^ female mice. *Pmch*^*ΔVglut2*^ male mice on HFD showed no difference from controls in total or percentage lean and fat mass, whereas *Pmch*^*ΔVglut2*^ females exhibited lower fat mass than controls on HFD (Fig. 6I-M).

Locomotor activity during dark phase, light phase, and across a 24 h in all three planes was similar between genotypes in both sexes (Fig. 6M-N, X-plane *p* = 0.99, Y *p* = 0.95 and Z *p* = 0.96 for females, and X-plane *p* = 0.76, Y-plane *p* = 0.44 and Z *p* = 0.86 for males, Sidak’s multiple comparisons test).

### Male *Pmch*^*ΔVglut2*^ mice on HFD showed improved insulin resistance

**Fig. 7:**
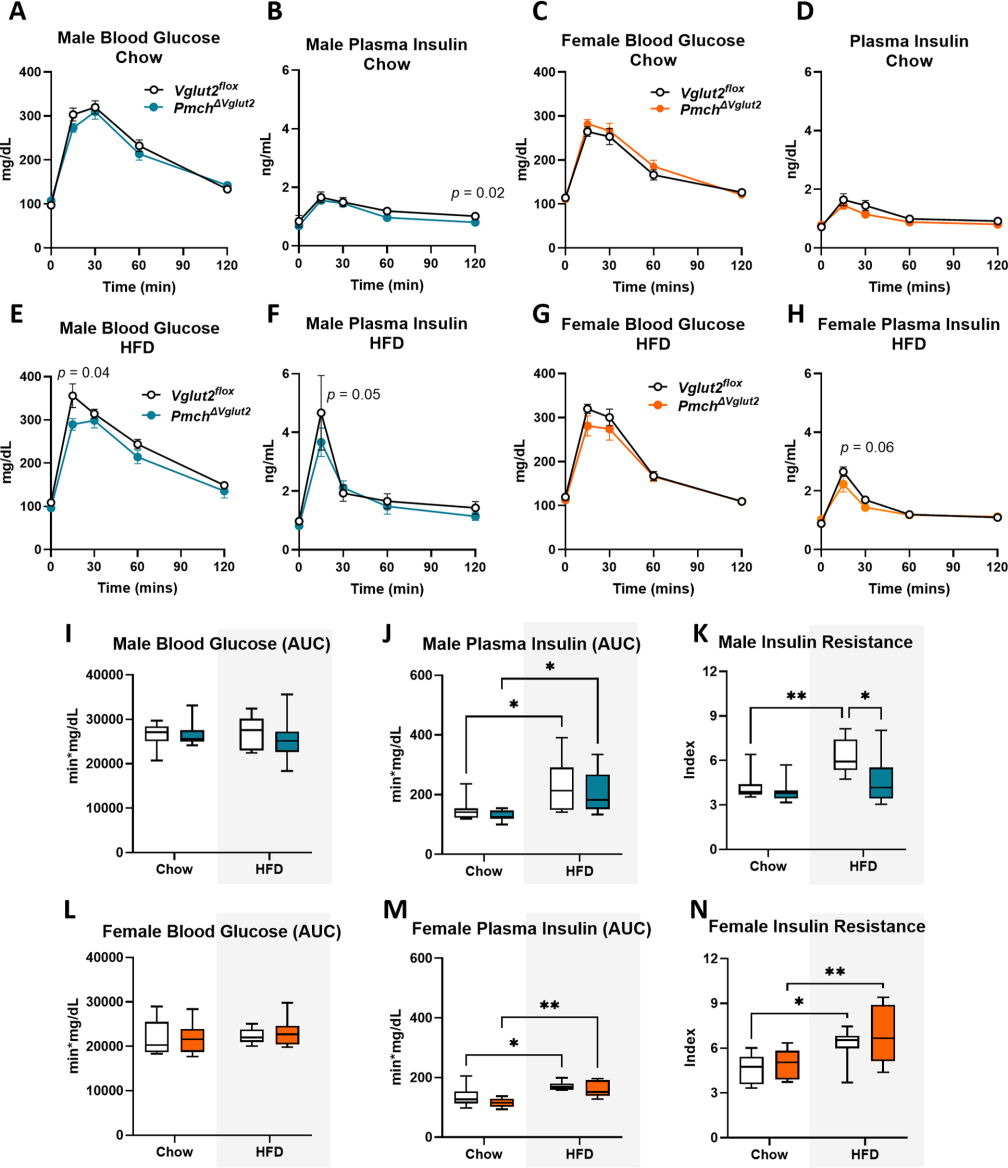
Male *Pmch*^*ΔVglut2*^ mice on high fat diet (HFD) showed improved insulin resistance. (**A-D**) Graphs showing glucose and insulin levels following glucose tolerance test (GTT) in males (A-B, *n* = 10 *Vglut2*^*flox*^, *n* = 11 *Pmch*^*ΔVglut2*^) and females (C-D, *n* = 8 *Vglut2*^*flox*^, *n* = 11 *Pmch*^*ΔVglut2*^) fed a regular chow diet. (**E-H**) Graphs showing glucose and insulin levels following GTT in males (E-F, *n* = 7 *Vglut2*^*flox*^, *n* = 9 *Pmch*^*DVglut2*^) and females (G-H, *n* = 7 *Vglut2*^*flox*^, *n* = 9 *Pmch*^*ΔVglut2*^) fed an HFD. (**I-J**,** L-M**) Box and whiskers graph showing area under the curve of graphs A-H comparing mice in regular chow diet and HFD. No change in glucose but increase in insulin levels were detected in both genotypes and both sexes on HFD. In J, * *p* < 0.025 df = 31 and in M, * *p* = 0.23 and ** *p* = 0.001, df = 31. (**K**,** N**) Box and whiskers graph showing index of insulin resistance in male (K) and female (N) on regular chow and HFD. Note that index of insulin resistance is lower in *Pmch*^*ΔVglut2*^ male mice compare to *Vglut2*^*flox*^ on HFD. This difference was not observed in *Pmch*^*ΔVglut2*^ female mice. In K, * *p* = 0.033 and ** *p* = 0.0085 df = 29. In N, * *p* = 0.023 and ** *p* = 0.0029 df = 31. Two-way ANOVA and Tukey’s multiple comparison test. Analysis performed in the MMPC in two trials with *n* = 4–6/sex and genotype every trial. Cohorts were combined for analysis (total *n* = 7–11/sex and genotype). Data from *Vglut2*^*flox*^ mice (experimental controls) is represented in white color and from *Pmch*^*ΔVglut2*^in blue for males and orange for females

Studies have shown that MCH neuronal ablation as well as conditional *Vglut2* deletion from MCH neurons can both improve glucose tolerance [16, 34]. Despite extensive documentation of sex differences in glucose handling [78–83] the effects of deletion of *Vglut2* in MCH neurons have not been investigated in female mice. Following metabolic phenotyping, male and female mice on regular chow and HFD (20–21 weeks of age) remained single housed and were tested for glucose tolerance. On a chow diet, neither male nor female *Pmch*^*ΔVglut2*^ mice showed any difference in fasting glucose levels, glucose tolerance, or insulin levels compared to controls (Fig. 7A-D). Sex differences were noticed, however, when mice were fed a HFD for 12 weeks. Whereas females from both genotypes showed no detectable changes in fasting glucose, glucose tolerance and insulin levels, *Pmch*^*ΔVglut2*^ male mice exhibited a slight improvement in glucose handling (time point: 15 min) (Fig. 7E-H), and a notable amelioration of insulin resistance when fed a HFD (Fig. 7I-K). In females, insulin resistance increased in same magnitude in both genotypes following 12 weeks of HFD (Fig. 7L-N, Supplementary Figure D).

## Discussion

We describe the generation and validation of a new transgenic knock-in mouse model for the study of the MCH neuronal function and circuitry. The *Pmch*-iCre mice express iCre recombinase driven by the *Pmch* promoter. Virtually all MCH neurons coexpress *Pmch*-iCre EGFP in the LHA, PFx and ZIm (IHy) in adult mice of both sexes, and the pattern of iCre-induced EGFP expression resembles that described in previous studies [5, 44]. Additional EGFP + sites were also observed, although whether they are a result of transient *Pmch* expression during development needs further investigation.

In late-lactation dams, iCre-induced EGFP expression aligns with lactation-induced *Pmch* mRNA expression in the mPOA and PVH [44, 61, 62, 74] indicating that the *Pmch*-iCre is a more suitable model for the study of the MCH system of female mice in diverse physiological states pertaining to the endocrine functions of MCH neurons. Using the new *Pmch*-iCre mouse line, we generated *Pmch*^*ΔVglut2*^ mice to fill in gaps in the existing literature. Specifically, we incorporated females into our experimental design and assessed reproductive and metabolic function in both sexes.

Support for MCH neuronal influence on reproductive physiology is contentious. MCH terminals are in close apposition with gonadotropin releasing hormone (GnRH) neurons, which express MCH receptor 1 (MCHR1) [84–88]. In acute hypothalamic slices from female mice, MCH application blocks kisspeptin activation of GnRH neurons, while in cultured hypothalamic explants, MCH induces release of GnRH [84–86, 89]. MCH injection into the female rat brain affects luteinizing hormone (LH) release in a manner which depends on both site of injection and the ovarian hormone milieu, though the precise relationship between these variables is not well understood [89, 90].

The rostral hypothalamic upregulation of MCH during lactation suggests a role therein; however, it seems that mPOA and LHA MCH neurons may have opposing functions in this regard [91, 92]. While MCH in the mPOA arises late in lactation and appears to be associated with the cessation of lactation and weaning of pups [91], stimulation of LHA MCH neurons increases nursing behavior [92] and systemic administration of an MCHR1 antagonist shortly postpartum reduces maternal behaviors and production of milk [93].

The functional role of glutamate from MCH neurons in reproductive physiology has not, to our knowledge, been systematically evaluated previously. The delay we observed in sexual maturation of *Pmch*^*ΔVglut2*^ female mice in the absence of changes to either body weight or fat mass suggests that glutamatergic neurotransmission contributes to the activation of the hypothalamo-pituitary-gonadal (HPG) axis during the pubertal transition. Whether this effect is attained via direct innervation of GnRH neurons warrants a more detailed investigation due to the aforementioned ability of MCH to inhibit GnRH neuronal activity via blockade of kisspeptin action in hypothalamic preparations [85].

While MCH-KO mice show altered estrous cyclicity, the *Pmch*^*ΔVglut2*^ mice exhibit regular estrous cycles even when challenged with HFD [68, 94] suggesting the lack of *Vglut2*_*ΔVglut2*_ mitigates cycle dysregulation. However, these mice display a significant increase in latency to pregnancy. The underlying neural substrate for this phenotype is uncertain, but it may be linked to olfactory disruption in *Pmch*^*ΔVglut2*^ mice. The MCH system targets brain sites associated with olfactory integration, and MCH-KO mice showed disruption of social behaviors driven by odorants, including mating, aggression and maternal behavior [93, 95–97]. Lack of glutamate in MCH neurons may lead to impairment in olfactory discrimination, affecting sexual receptivity and mating behavior resulting in the increased latency to pregnancy observed in *Pmch*^*ΔVglut2*^ mice.

The role of MCH in metabolism has been assessed in many ways. For example, targeted expression of human diphtheria toxin receptor (DTR) to the *Pmch* gene and the resultant ablation of MCH neurons following diphtheria toxin injection produced a lean, hypophagic, hyperactive phenotype [15]. In such cases, where MCH-KO recapitulates MCH neuronal ablation, it seems to indicate that MCH is the critical neuromodulator driving those changes [15, 16]. However, there is also evidence for functional redundancies between MCH and glutamatergic signaling from MCH neurons. Characterization of the metabolic phenotypes of male MCH neuron-ablated, MCH-KO, and transgenic *Pmch*-Cre;*Slc17a6fl/fl* mice (which lack VGLUT2 in MCH neurons) showed that all three experimental groups exhibit decreased body weight and adiposity, increased locomotor activity, late-onset hypophagia, attenuated weight gain on high-fat diet, and reductions in circulating leptin [15, 16].

Similar to what has been reported using the commercially available *Pmch*-Cre mouse models [15, 16] our *Pmch*^*ΔVglut2*^ showed late onset leanness on a standard chow diet. When challenged with a HFD, *Pmch*^*ΔVglut2*^ mice were protected from excessive weight gain. On a regular chow diet, mice of both sexes display a strong diurnal pattern in both food consumption and locomotor activity, with most of the feeding and activity occurring during the dark phase. HFD is a well-documented disruptor of feeding patterns, with changes observed after one week of HFD consumption, before weight gain is detected [77]. While the *Vglut2*^*flox*^ control mice showed an altered diurnal pattern of food consumption on an HFD, *Pmch*^*ΔVglut2*^ mice exhibited no detectable changes in their diurnal rhythm of food intake. Of note, mice lacking *Vglut2* in MCH neurons also exhibit abnormal REM sleep patterns [19]. The circuits and mechanisms underlying these effects are unknown, although MCH neurons project to the suprachiasmatic nucleus—the body’s “master clock”— and their ablation alters circadian periodicity and behavioral rhythms [98].

Still other studies have highlighted the unique contributions of MCH and glutamate in glucose homeostasis. Improved glucose handling has been reported in mice with MCH neuronal ablation and conditional *Vglut2* deletion in MCH neurons, but not in global MCH-KO [15, 16] suggesting a glutamate-dependent mechanism by which MCH neurons contribute to glucose homeostasis. Our findings partially support this interpretation, as improved insulin resistance was observed in *Pmch*^*ΔVglut2*^ male mice. However, this effect was not observed in *Pmch*^*ΔVglut2*^ females. This finding is highly relevant to diabetes research because sex differences in glucose handling are extensively described in rodents and primates, including humans, but the underlying mechanisms are unknown [20–22]. It is worth noting that estrogens enhance MCH neuronal sensitivity to glucose while androgens appear to depress it, which could be masking an effect of VGLUT2 deletion in female mice [99].

While most findings in male mice were reproduced using the new *Pmch*^*ΔVglut2*^ mice, some inconsistencies, such as lack of change in locomotor activity, were observed. This should be interpreted in the context of documented background strain effects on various aspects of metabolic function, susceptibility to obesity and type II diabetes [100–104]. For instance, overexpression of MCH causes increased body weight on a C57BL/6 background, but it does not do so on an FVB background [9]. Indeed, mouse MCH knockout models show substantial phenotypic differences based on the background strain [10]. At baseline, male C57BL/6 mice are susceptible to diet induced obesity and glucose intolerance, while 129 mice are comparatively resistant to such phenotypes. MCH knockout results in body weight reduction on a C57BL/6 compared to a 129 background despite inducing a proportionally greater increase in spontaneous motor activity on a 129 background [10]. Where background strain differences have been noted, studies of conditional *Vglut2* deletion in MCH neurons have used mice on a C57BL/6 background [85]. The *Pmch*^*ΔVglut2*^ mice in this study were in a mixed C57BL/6 and 129 background.

Overall, our data show that, on a mixed genetic background, conditional deletion of *Vglut2* in MCH neurons has sex-specific effects on pubertal development and response to HFD including weight gain and insulin resistance. Our findings also indicate that glutamatergic neurotransmission from MCH neurons is associated with the disruption of daily pattern of food intake in mice on HFD, which is itself intimately tied to metabolic disease: circadian misalignment of feeding contributes to the development of obesity as well as associated cardiometabolic disorders. These results strongly support the necessity of including female subjects for the study of the MCH system in metabolism, reproduction, and beyond.

## Supplementary Information

Below is the link to the electronic supplementary material.

Supplementary Material

## Data Availability

No datasets were generated or analysed during the current study.
