# Supplementary figures and images for: Lack of glutamate neurotransmission in melanin-concentrating hormone neurons alters mouse reproduction and metabolism in a sex-specific manner
Source: Biol Sex Differ. 2025 Aug 6;16:59. doi: 10.1186/s13293-025-00742-3 (PMC12326619; doi:10.1186/s13293-025-00742-3)

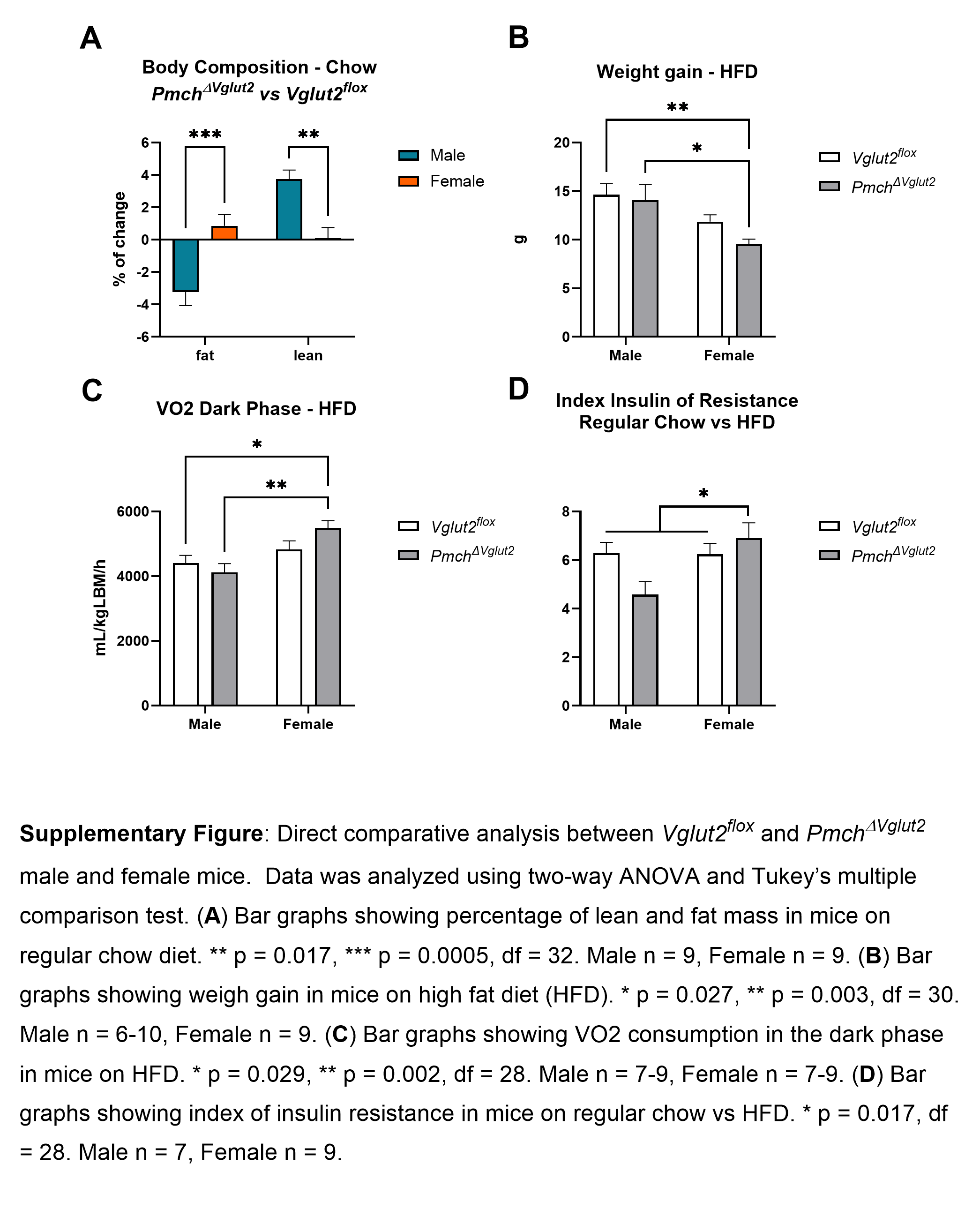

Supplement: Supplementary file 1 — Supplementary Material [file 13293_2025_742_MOESM1_ESM.tif]
